# Automatic Recognition of Colon and Esophagogastric Cancer with Machine Learning and Hyperspectral Imaging

**DOI:** 10.3390/diagnostics11101810

**Published:** 2021-09-30

**Authors:** Toby Collins, Marianne Maktabi, Manuel Barberio, Valentin Bencteux, Boris Jansen-Winkeln, Claire Chalopin, Jacques Marescaux, Alexandre Hostettler, Michele Diana, Ines Gockel

**Affiliations:** 1 Institute for Research against Digestive Cancer (IRCAD), 67091 Strasbourg, France; manuel.barberio@ircad.fr (M.B.); jacques.marescaux@ircad.fr (J.M.); alexandre.hostettler@ircad.fr (A.H.); michele.diana@ircad.fr (M.D.); 2 Innovation Center Computer-Assisted Surgery (ICCAS), University of Leipzig, 04103 Leipzig, Germany; Marianne.Maktabi@medizin.uni-leipzig.de (M.M.); claire.chalopin@medizin.uni-leipzig.de (C.C.); 3 General Surgery Department, Card. G. Panico, 73039 Tricase, Italy; 4 ICUBE Laboratory, Photonics Instrumentation for Health, University of Strasbourg, 67400 Strasbourg, France; valentin.bencteux@ihu-strasbourg.eu; 5 Department of Visceral, Transplant, Thoracic and Vascular Surgery, University Hospital of Leipzig, 04103 Leipzig, Germany; boris.jansen-winkeln@medizin.uni-leipzig.de (B.J.-W.); ines.gockel@medizin.uni-leipzig.de (I.G.); 6 Department of General, Digestive, and Endocrine Surgery, University Hospital of Strasbourg, 67091 Strasbourg, France; 7 INSERM, Institute of Viral and Liver Disease, 67091 Strasbourg, France; 8 Mitochondrion, Oxidative Stress and Muscle Protection (MSP)-EA 3072, Institute of Physiology, Faculty of Medicine, University of Strasbourg, 67085 Strasbourg, France

**Keywords:** hyperspectral imaging, machine learning, convolutional neural networks, cancer, computer-assisted diagnosis, image-guided surgery

## Abstract

There are approximately 1.8 million diagnoses of colorectal cancer, 1 million diagnoses of stomach cancer, and 0.6 million diagnoses of esophageal cancer each year globally. An automatic computer-assisted diagnostic (CAD) tool to rapidly detect colorectal and esophagogastric cancer tissue in optical images would be hugely valuable to a surgeon during an intervention. Based on a colon dataset with 12 patients and an esophagogastric dataset of 10 patients, several state-of-the-art machine learning methods have been trained to detect cancer tissue using hyperspectral imaging (HSI), including Support Vector Machines (SVM) with radial basis function kernels, Multi-Layer Perceptrons (MLP) and 3D Convolutional Neural Networks (3DCNN). A leave-one-patient-out cross-validation (LOPOCV) with and without combining these sets was performed. The ROC-AUC score of the 3DCNN was slightly higher than the MLP and SVM with a difference of 0.04 AUC. The best performance was achieved with the 3DCNN for colon cancer and esophagogastric cancer detection with a high ROC-AUC of 0.93. The 3DCNN also achieved the best DICE scores of 0.49 and 0.41 on the colon and esophagogastric datasets, respectively. These scores were significantly improved using a patient-specific decision threshold to 0.58 and 0.51, respectively. This indicates that, in practical use, an HSI-based CAD system using an interactive decision threshold is likely to be valuable. Experiments were also performed to measure the benefits of combining the colorectal and esophagogastric datasets (22 patients), and this yielded significantly better results with the MLP and SVM models.

## 1. Introduction

### 1.1. Clinical Context

There are approximately 1.8 million diagnoses of colorectal cancer each year globally and 0.6 million diagnoses of esophageal cancer with a high mortality (sixth rank among all cancer types). There are approximately 1 million diagnoses of stomach cancer reaching the third rank in terms of mortality [1]. An early diagnosis of these cancer types is essential to reduce mortality rates and improve treatment options, including surgery. Endoscopy is crucial to detect these cancers and other abnormalities in tissues [2]. Accurate diagnosis and staging are fundamental before considering a possible therapeutic strategy. Computer-assisted diagnostic (CAD) tools are becoming increasingly important to reduce human subjectivity and cost and to facilitate earlier diagnosis.

CAD tools have great potential to help gastrointestinal endoscopists in various ways [3], namely to help locate precancerous and cancerous tissue and to ensure negative margins after the resection of a lesion, hence, allowing for a more accurate diagnosis and globally improving the oncological outcomes. However, the existing CAD tools for endoscopic detection of colorectal and esophagogastric cancerous tissues are based exclusively on features recognized on conventional RGB pictures, which poses obvious limitations. CAD tools allowing for an automatic endoscopic cancer detection, based on imaging modalities, which can detect features beyond the human eye, could be more powerful and exhibit increased diagnostic accuracy.

### 1.2. Hyperspectral Imaging and Medical Applications

Hyperspectral imaging (HSI) is an optical imaging technique that is gaining popularity in medical image analysis and CAD [4,5,6,7]. HSI is a non-invasive and relatively inexpensive imaging technology that uses a broadband light source to measure optical tissue properties across different electromagnetic bands. The light interaction (scattering of photons) of tissue is measured to generate spectral images at narrow spectral bands (typically generating 100 or more images). Each image measures the relative light absorbance or reflectance for a band that can reveal biological properties, such as chromophores and tissue oxygenation. This data is assembled into a discrete 3D volume with two spatial dimensions and one spectral dimension, called a hyperspectral image, hyperspectral cube or hypercube.

One main limitation of HSI is that the hypercube cannot be immediately interpreted by physicians. Machine learning (ML) is an essential component to learn and automatically recognize and convey relevant spatio-spectral patterns [8]. In the broader field of CAD, HSI has been investigated to improve the diagnosis and resection of various types of cancerous lesions. It has been applied to detect gastrointestinal cancer [9,10,11,12,13,13], tongue cancer [14] oral cancer [15], skin tumors [16] breast cancer [17], and brain cancer [18]. All of these approaches use supervised learning, where regions of cancer and healthy tissue are identified in the training dataset and paired with the histopathology analysis. Supervised methods can be divided into two groups: traditional (also called the classical method) and deep learning methods.

Traditional methods include SVMs [9,19], Random Forest [20] and K-nearest-neighbor (KNN) [4]. Recently, deep learning methods, and particularly convolutional neural networks (CNNs) have been considered [21,22,23]. These have the advantage of automatically learning relevant spectral or spatio-spectral features encoded in convolutional filters, reducing the need and human effort for designing handcrafted features. However, their main limitation is that they usually require significantly more data for adequate generalization. In medical applications, the requirement for large annotated datasets to train such models is one of the greatest limiting factors to clinical translation. There are also difficulties specific to HSI, which make it difficult to acquire sufficient and representative data. Currently, HSI is not used in the standard of care. Consequently, the acquisition of data requires specific experimental protocols. In addition, a high inter-patient variability is common [24], making fine-grained diagnoses, such as cancer type differentiation, difficult.

Today, there is an undeniable momentum to translate HSI CAD systems into clinical use. Recently, HSI systems for open surgery have now become commercially available [25]. To significantly expand the use of HSI in surgery, there is ongoing effort to miniaturize HSI equipment for use in minimally invasive surgery. The first laparoscopic system with a high-resolution color video and simultaneous HSI with a high spatial and spectral resolution ranging from 500 to 1000 nm was presented in [26]. For use in gastroenterology, flexible endoscopic prototype systems have recently been proposed [19,27,28,29].

### 1.3. Contribution Summary

There have been some previous works for automatic colorectal cancer tissue detection with HSI and ML [9,10,19,30,31]. Support vector machines (SVMs) have been used with ex vivo samples for colon cancer detection [9] and in vivo with a modified laparoscopic system using multispectral imaging [10]. Other classical models, including linear discriminant analysis (LDA), have been considered [30]. A flexible HS colonoscopy system was used to test in vivo colorectal cancer recognition with SVMs [19]. There is only one previous work that demonstrated the use of HSI to detect esophagogastric cancer with human data [13]. In this study, classical ML models were evaluated to identify tumors in extraluminal HSI. The current work differs in the fact that intraluminal tissue is imaged.

Additionally, a major open challenge to translate such ML models into routine clinical practice is the lack of large annotated HSI datasets. We explored the possibility to enlarge training datasets by combining hypercubes of different cancer types (colonic and esophagogastric). We trained ML models to automatically differentiate healthy mucosa from cancer, without attempting to differentiate the cancer or tissue types. This differentiation is unnecessary regarding surgical guidance for tumor resection, and it allowed us to train our models with substantially more data. We showed that, by combining datasets, the performance of classical ML models could be significantly improved.

We also showed that the test performance of all models could be significantly improved by tuning their classification detection thresholds at test time. We called this *patient-specific decision threshold tuning*. In all prior HSI-based cancer detection and classification with machine learning, fixed detection thresholds were used, and we showed that this gave a substantially worse performance with standard metrics, such as the Sorensen–Dice coefficient (commonly referred to as DICE). This finding has opened the opportunity to improve results with patient-specific decision threshold fine-tuning at test time. Finding the optimal patient-specific decision threshold is not trivial, however, and it is an important technical challenge that requires future work that is justified by the findings of our study.

## 2. Material and Methods

### 2.1. Data Collection and Annotation

The clinical study was performed at the University Hospital of Leipzig using a dataset of 10 patients with esophagogastric cancer and 12 patients with colon cancer. For each patient, one hypercube was captured using the TIVITA system by Diaspective Vision GmbH, Pepelow, Germany (Table 1 and Table 2). As a result, the dataset had 22 hypercube with one hypercube per patient. The study was approved by the local ethics committee of the medical Faculty of the University of Leipzig (026/18-ek), the study was also registered at Clinicaltrials.gov (NCT04230603) (accessed on 18 July 2021). Resected tissue samples were imaged under standardized conditions during the surgical procedure within 5 min after resection. Specifically, esophageal and colonic tracts were cut lengthwise and opened, and the inside mucous tissue was imaged.

There was no ambient light, the HS camera was located at 50 cm from the sample (i.e.,  the calibrated distance), and a spectral range of 500 to 1000 nm was used. Acquisition time was approximately 10 s. Two different objectives were used with image sizes 280×210 mm and 80×65 mm. These had a spectral resolution of 640 × 480 pixels (0.44 mm/pixel and 0.13 mm/pixel, respectively). Standard normalization preprocessing was applied to each hypercube (Standard Normal Variate [32]) to reduce the variability from tissue surface orientation and light scattering effects, for instance. An experienced pathologist and the surgeon then annotated the RGB image (provided with the device and simulated from the hypercube) based on the comparison with the histopathological slides, with regions of interest (ROIs) and interactive software (Gimp v. 2.10). ROIs for four classes were annotated.

**Figure 1:**
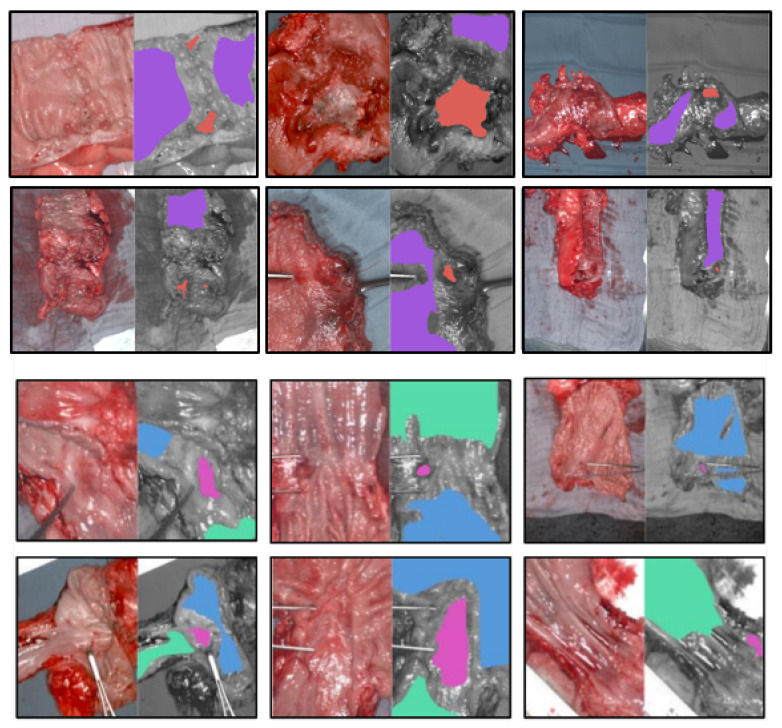
Example annotated images from the colon and esophagogastric datasets. The top two row shows RGB images of colon samples with associated annotations side-by-side: purple annotation is for healthy colon tissue, red is for cancer tissue. The bottom row shows stomach and esophagus samples: green annotation is for healthy esophagus tissue, blue for healthy stomach tissue and pink is for cancer tissue.

One class corresponded to malignant tissue, macroscopically corresponding to the center of the tumor. The other three classes were healthy mucosa of colon, stomach, and esophagus, respectively. We showed representative images from the dataset in Figure 1. RGB images synthesized from 10 hypercubes are shown side-by-side with the corresponding spatial annotations. There is a clear imbalance in the dataset where the healthy mucosa is considerably more represented than the cancer tissue. Patients 2 and 6 of the colon dataset did not have cancer. As a result, only healthy tissue annotations were made for those patients. We summarized the colon and esophagogastric datasets in Table 1 and Table 2.

### 2.2. Dataset Analysis with Spectral Curves

**Figure 2:**
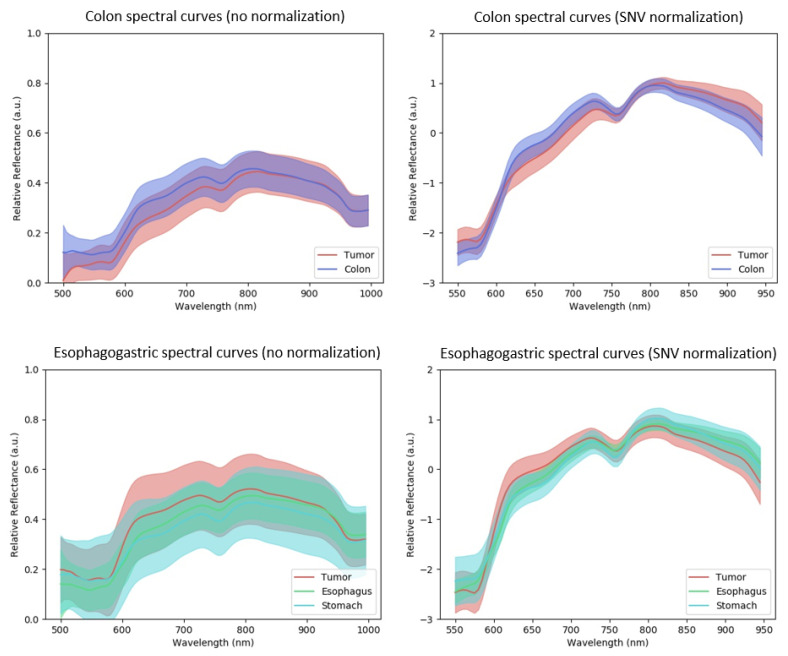
Spectral curves for colon and esophagogastric datasets with and without SNV normalization. Solid curves show the mean spectral curves, and filled regions show 1 standard deviation from the mean curve.

The intrinsic difficulty of tissue classification can be gauged by visualizing spectral curve distributions. Figure 2 shows spectral curves corresponding to the colon (a, b) and esophagogastric datasets (c, d). In each sub-figure, we have shown mean spectral curves for cancer and healthy tissue classes. Intra-class variability is represented as solid bands with a width of ±standard deviation from the mean spectral curve. Spectral curves are shown with and without SNV normalization, (a, c) and (b, d), respectively. For the colon dataset (a), we observe relatively low intra-class variability with strong overlap in spectral curves, indicating a difficult classification problem.

With SNV normalization (b) we observed a narrower intra-class variability, which was expected. However, we also observed a general increase in inter-class variability. This suggested that SNV normalization might improve classification performance. For the esophagogastric dataset we observed a strong class overlap. However, one could generally observe separation between cancer and healthy tissue without normalization compared to the colon dataset (650 to 950 nm).

### 2.3. Models, Training Processes and Performance Metrics

#### 2.3.1. Classical Machine Learning Models

We trained and tested the classical ML models applied to HSI data: Random Forest, logistic regression, multilayer perceptron (MLP) and SVM with linear and radial basis functions (RBFs). The class imbalance existing in our datasets was handled by random downsampling. Specifically, the number of samples S from the least represented class was evaluated, and all other classes were randomly downsampled (uniform probability without replacement) to have S samples. SNV normalization was used. The classical ML models were implemented using Python’s scikit-learn library [33]. We used a log-loss function and a limited Broyden–Fletcher–Goldfarb–Shanno solver. Our activation function was a hyperbolic tangent and two hidden layers were implemented.

#### 2.3.2. Convolutional Neural Network Models

CNNs have been shown to perform appropriately for the segmentation and classification of hyperspectral data in spite of limited data [34,35]. To handle limited data, the best performing methods operated by dividing the hypercube into spatially localized sub-volumes where each sub-volume was processed independently. This had two main benefits.

The first was to significantly reduce the size of the CNN and its number of trainable parameters compared to a much larger CNN for processing the full hypercube at once. The second benefit lay in the fact that that the CNN could be trained with many sub-volumes extracted from the same hypercube, which reduces overfitting. State-of-the-art CNNs can be broadly divided into 1DCNNs, such as Hu et al. [36], which use a spatial window of only one pixel, and 3DCNNs that use a square or rectangular spatial window of more than one pixel [37,38,39,40].

The advantages of 3DCNNs are to exploit spatio-spectral features to improve classification performance. We tested the 3DCNN of Hamida et al. [40] (version d), which was shown to perform well for classifying remote sensing hyperspectral data with small datasets. We refer the reader to Hamida et al. [40] for precise network architecture details. The 3DCNN took a hypercube sub-volume of size 5×5×L (a spatial window of 5 × 5 pixels and *L* frequency channels with L=100) as input.

The 3DCNN was structured in a series of encoder layers that extracted spatial-spectral features of different scales using a series of 3D and 1D convolutional layers with max pooling. The design was inspired by SqueezeNet [41] that decomposed 3D convolutions into sequences of 1D convolutions, which also reduced the number of weights significantly. The final layer was fully connected with *N* output neurons where *N* denotes the number of classes. We used the 3DCNN for binary classification with N=2, and it has 32,232 trainable weights. We emphasize that this was a relatively light CNN with far fewer parameters than common CNNs for image segmentation, such as U-Net [42] or its variants that can have millions of trainable weights. This allowed our 3DCNNs to be trained on a relatively small cohort.

#### 2.3.3. Leave-One-Patient-Out Cross Validation (LOPOCV)

As the dataset size was limited, all models were trained and tested using the leave-one-patient-put cross-validation (LOPOCV) strategy. LOPOCV measures the generalizability of the models to novel patient data, and it is standard practice to evaluate ML models in medical applications with limited datasets. As one hypercube was acquired per patient, LOPOCV was implemented by cycling through each hypercube, training the model on all remaining hypercubes, and testing performance on the held-out hypercubes. This process was repeated so that each patient’s hypercube was held out and used for testing. We noted that LOPOCV has not been consistently used in HSI research. Performance has often been evaluated by testing and training the model on the same hypercube [9,30,43]. This severely inflates performance and it does not reflect the real expected performance of the model on new patient data.

#### 2.3.4. Evaluation with the Colon, Esophagogastric and Combined Datasets

We evaluated each model in three settings. In the first setting, we trained and tested the performance with LOPOCV using images from the colon dataset. This has 12 images in total. Ten of the images had cancer and healthy colon tissue annotations, and 2 of the images (patients 2 and 6) had only healthy colon annotations, and there was no cancer tissue. In the second setting, we trained and tested the performance with LOPOCV using images from the esophagogastric dataset. All images of the esophagogastric dataset have both cancer and healthy esophagogastric tissue annotations. In the third setting, we trained and tested performance with LOPOCV by combining images from the colon and esophagogastric dataset.

We referred to this as the *combined* dataset with 22 images in total. In the combined dataset, we combined healthy colon and esophagogastric tissue into one class (the negative class), and we combined colon and esophagogastric cancer into a second class (the positive class).

#### 2.3.5. Training Implementation Details

The 3DCNN was implemented in Pytorch 1.4.0, and it was trained for each LOPOCV fold as follows. Kaiming initialization was used to randomly initialize the network weights and the network biases were initialized to zero. The network was trained by minimizing class-weighted binary cross-entropy with Stochastic Gradient Descent (SGD). This was implemented using Pytorch’s torch.optim package with a learning rate of 0.01 and a weight decay of 0.0005. Class weighting was implemented using inverse-frequency weighting to overcome the fact that the healthy class was significantly more represented than the cancer class in the training dataset.

Training was run for 200 epochs. At each epoch, batches of size 3000 were used where each batch consisted of 3000 randomly selected sub-volumes from all training images drawn with uniform probability without replacement. Training of one LOPOCV fold took approximately 7 h for the colon dataset, 8 h for the esophagogastric dataset and 15 h for the combined datasets. In addition to using a relatively small 3DCNN, to further reduce the risk of overfitting, a validation set was used, which is standard practice to avoid overfitting a CNN. Specifically, a random subset of 10% of the training pixels were used in the validation set. After each training epoch, the validation set loss was evaluated, and after all training epochs, the network weights with the lowest validations set loss were used as the final network weights.

## 3. Results and Discussion

### 3.1. Evaluation Metrics

We evaluated performance using three well-established metrics. The first metric is Receiver Operator Curve Area-Under-Curve (ROC-AUC). ROC-AUC gave the probability that a random positive example (cancer) was scored higher with a model as compared to a random negative example (healthy tissue). ROC-AUC was chosen because it summarized recognition performance with a single number that was not tied to a specific decision threshold. The second metric was the Matthews correlation coefficient (MCC). This measured the correlation between a model’s predicted classifications as compared to ground truth, with a value ranging from −1 and +1, where a higher value indicated better performance.

The third evaluation metric was the Sorensen–Dice coefficient (DICE), also called the F1 score, which measured the harmonic mean of precision and recall. We used both MCC and DICE because they were used extensively in the literature on machine learning HSI classification. Unlike ROC-AUC, MCC and DICE use a decision threshold defined for each model. We tested two policies in order to specify the threshold. The first policy used a *patient-generic threshold*, where for each model, a single threshold was used for all patients, for each model. The selected threshold was the one that maximized the model’s mean metric (MCC or DICE) on all test images. The second policy used a *patient-specific threshold*, where a different threshold was tuned for each patient. This threshold was found as the one that maximized the metric (MCC or DICE) for each patient’s image using grid-search.

Performance with a patient-specific threshold would generally be greater than performance with a patient-generic threshold. In clinical application settings, we cannot usually tune a patient-specific threshold automatically, as this would require knowing the true tissue class labels. Nevertheless, we compared performance with a patient-specific threshold to reveal the performance gap that existed between using a patient-specific and patient-generic threshold, and to see if the gap was similar across models.

### 3.2. Statistical Tests

Paired two-tailed t-tests were used to assess whether the difference in mean ROC-AUC scores from two models were significantly different. Colon patients 2 and 6 were excluded in the statistical analysis because they did not have cancer tissue, and therefore they do not have ROC-AUC scores. p≤0.05 was considered statistically significant.

### 3.3. ROC-AUC Results

ROC-AUC performance is shown in Table 3 and Table 4. In Table 3, columns 2, 3 and 4, we show the ROC-AUC performance for the RBF-SVM, MLP, and 3DCNN models when trained and tested on the colon dataset using LOPOCV. In Table 3, columns 5, 6 and 7, we show ROC-AUC performance of the models when trained on the combined dataset and tested on the colon dataset using LOPOCV. We also include the mean ROC-AUC and standard deviation in the bottom row.

From Table 3, we can see that the 3DCNN performed better than RBF-SVM and MLP, with a higher mean ROC-AUC and a lower standard deviation. We observed this both when the models were trained with the colon dataset and the combined dataset. We did not see any significant performance difference for any model when they were trained on the colon or combined datasets and tested on the colon dataset (p=0.89 for RBF-SVM, p=0.78 for MLP and p=0.84 for 3DCNN). There was no statistically significant difference between the 3DCNN, RBF-SVM and MLP models that were trained on the combined dataset.

In Table 4, columns 2, 3 and 4, we show the ROC-AUC performance for the RBF-SVM, MLP and 3DCNN models when trained and tested on the esophagogastric dataset using LOPOCV. The 3DCNN performed the best with a significant difference detected between MLP versus 3DCNN (p=0.030) and RBF-SVM versus 3DCNN (p=0.019) with the esophagogastric dataset. In Table 4, columns 5, 6 and 7, we show the ROC-AUC performance for the models when trained on the combined dataset and tested on the esophagogastric dataset using LOPOCV.

There was a substantial improvement in the RBF-SVM and MLP models, with mean ROC-AUC approaching that of 3DCNN. This indicated that the RBF-SVM and MLP models had significantly benefited from the additional trained data provided with the colon cancer images. In contrast, the performance of the 3DCNN was slightly improved when it was trained on the combined dataset. The RBF-SVM improvement was significant with p=0.0046. The improvement of the other models was not significant (p=0.074 for the MLP and p=0.22 for the 3DCNN models).

The relatively high ROC-AUC scores from the 3DCNN indicate that it performed very well at ranking healthy and tissue tumor classes, with a mean ROC-AUC above 0.90 in the colon and esophagogastric datasets. A ROC-AUC above 0.9 is generally considered an excellent result [44]. Of note, a ROC-AUC of 0.90 is equivalent to saying that a model has a 90% chance of attributing a higher classification score to cancer tissue as compared to healthy tissue. Unlike the RBF-SVM and MLP models, the 3DCNN model did not appear to benefit from enlarging the number of training images by combining the datasets.

We further investigated the benefit of training models by combining datasets with a cross-dataset training experiment. Specifically, we trained the MLP and 3DCNN models on the colon dataset, and we tested them on the esophagogastric dataset. We then repeated this process by training the models on the esophagogastric dataset and tested them on the colon dataset. The purpose of this experiment was to investigate whether the models could classify cancerous and healthy tissue significantly better than chance. If so, then it would tell us that there might be useful hyperspectral information that could be exploited for training a model with data from a different tissue and cancer type. We present the results in Table 5 where we report the ROC-AUC performance.

We observed that the 3DCNN achieved a mean ROC-AUC of 0.78 when trained on the esophagogastric dataset and tested on the colon dataset. It achieved a mean ROC-AUC of 0.86 when trained on the colon dataset and tested on the esophagogastric dataset. This was indeed an unusual result. Of note, the 3DCNN model achieved a mean ROC-AUC of 0.91 when trained and tested on the esophagogastric dataset, which was only marginally better than when it was trained on the colon dataset. This strongly indicated that there was useful hyperspectral information for training a model with data from a different tissue and cancer type. Performance of the MLP was also significantly better than random guessing (equivalent to a ROC-AUC of 0.5); however, its performance was far behind the 3DCNN.

### 3.4. MCC and DICE Results

In Table 6, we present the mean MCC and standard deviation for each model trained either with the colon, esophagogastric or combined datasets. We divided the table into MCC performance using a patient-generic and patient-specific decision threshold (see Section 3.1). We first considered test results on the esophagogastric dataset. Both the RBF-SVM and MLP models improved performance when trained on the combined dataset as compared to training on the esophagogastric dataset. This was true when we used a patient-generic or a patient-specific decision threshold. In contrast, for the 3DCNN model, we did not see any similar performance improvement. Indeed, its performance was slightly worse when it was trained on the combined dataset. For all models, the performance was much better using a patient-specific decision threshold compared to a patient-generic threshold.

This implied that it is difficult to obtain a single decision threshold that worked well for all test images. We could see very similar performance trends for the DICE metric as with the MCC metric in Table 7: The 3DCNN performed significantly better than the other models without training on the combined dataset. When training on the combined dataset, much better results were obtained by the RBF-SVM and MLP models for the esophagogastric dataset. In contrast, those models had very similar performance on the colon dataset when they were trained with the colon or combined datasets.

### 3.5. Results Visualization

**Figure 3:**
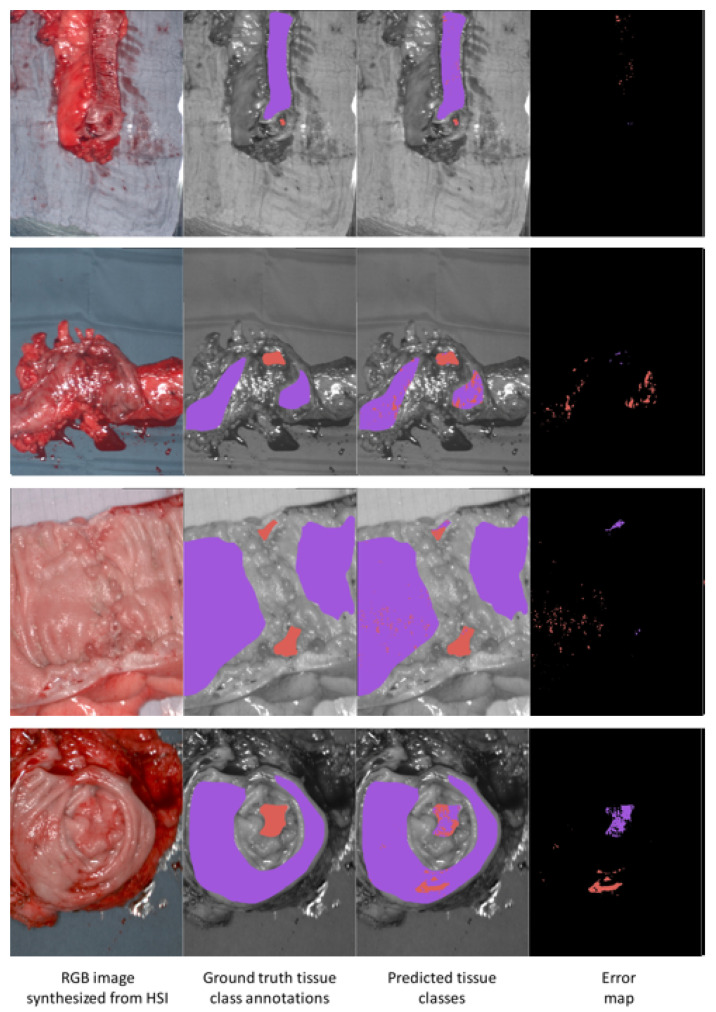
Visualization of results of the 3DCNN model on the colon dataset. Each row of images represents a patient (from top to bottom, patients 4, 3, 9 and 5). Each row of images shows four images. From the left, these are: (1) The RGB image synthesized from the hypercube. (2) The ground truth tissue class annotations provided by the surgeon. (3) The predicted tissue classes predicted by the 3DCNN. (4) The error map. Colored pixels in the error map indicates pixels where the 3DCNN prediction differed from ground truth. The displayed color is the color of the incorrect prediction from the 3DCNN. Pixels in red correspond to the tumor class, and pixels in purple correspond to the healthy tissue class.

**Figure 4:**
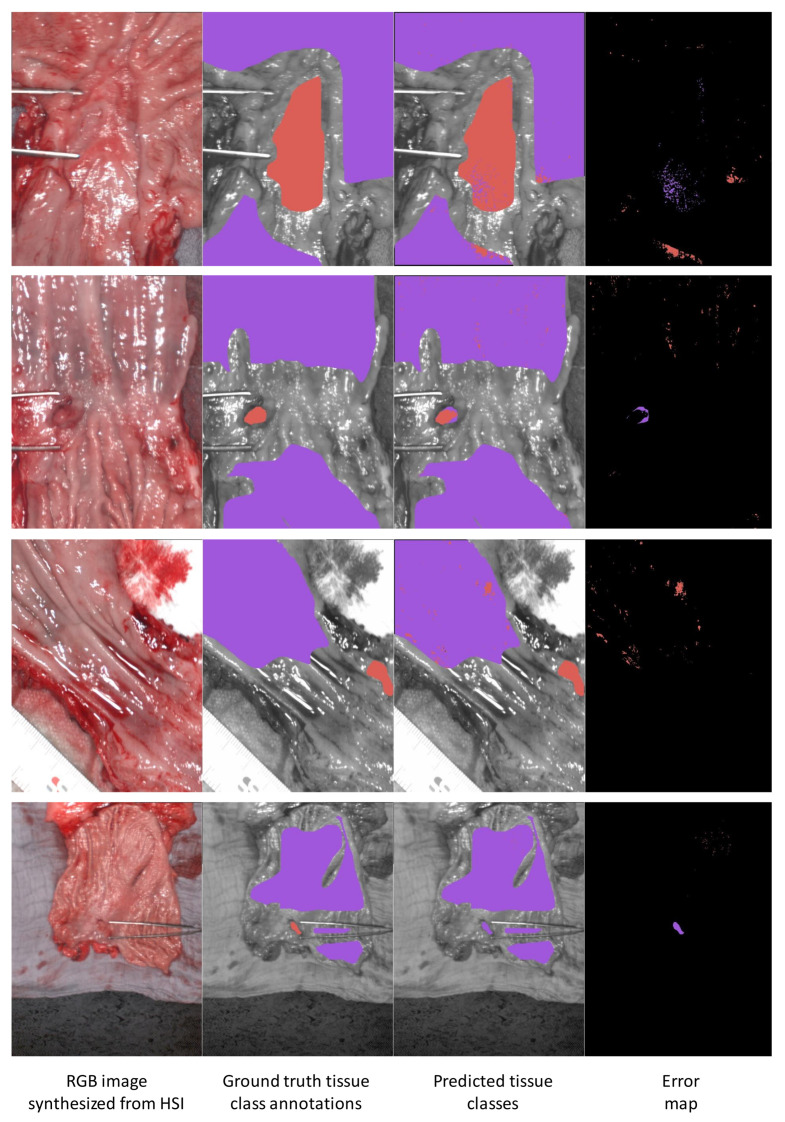
Visualization of results of the 3DCNN model on the esophagogastric dataset. Each row of images represents a patient (from top to bottom, patients 21, 19, 14 and 20). Each row of images shows four images. From the left, these are: (1) The RGB image synthesized from the hypercube. (2) The ground truth tissue class annotations provided by the surgeon. (3) The predicted tissue classes predicted by the 3DCNN. (4) The error map. Colored pixels in the error map indicates pixels where the 3DCNN prediction differed from ground truth. The displayed color is the color of the incorrect prediction from the 3DCNN. Pixels in red correspond to the tumor class, and pixels in purple correspond to the healthy tissue class.

We visualized the results from the 3DCNN model for the colon and esophagogastric datasets in Figure 3 and Figure 4, respectively. In each figure, results with four different patients are shown. In Figure 3 we observed that healthy tissue pixels were generally correctly classified in all images, as is the tumor class in patients 4 and 3 (rows 1 and 2). For patient 9, there were two regions in the image that corresponded to the tumor class and both regions were mostly correctly classified by the 3DCNN. For patient 5, the 3DCNN failed to correctly classify the tumor.

Regarding the esophagogastric dataset shown in Figure 4, we observed that healthy tissue pixels were also generally well classified in all images, as was the tumor class in patients 21, 19 and 14 (rows 1, 2 and 3). For patient 20, the 3DCNN failed to correctly classify the tumor. The fact that the tumor regions were relatively small could partially explain the low DICE and MCC scores, because a small number of false positives had a large influence on the DICE and MCC coefficients.

### 3.6. Discussion

HSI is emerging as a powerful and non-invasive imaging modality for detecting and diagnosing cancer [4,24]. Cancer tissue is associated with morphological and biochemical alterations that lead to changes in the tissue’s optical properties, in particular how light is reflected, absorbed, scattered and emitted as it passes through tissue [4,6]. Light scattering is related to the tissue microstructure, and absorption is related to the molecular composition. An HSI camera measures an aggregate signal of these optical properties in specific light wavelength bands in the NIR and visible light range, generating spectral profiles (also called spectral signatures [45]) of healthy and diseased tissue.

The HSI camera used in this study has wavelengths bands in the range of 500 to 1000 nm. In this range, the spectral profile can be understood at the molecular level. The spectral profile of hemoglobin (Hb) is very different in its oxygenated and deoxygenated states, and it strongly contributes to the overall spectral profile of tissue [46]. Changes in metabolic activity and physiology, such as angiogenesis, are associated with cancer [47], which alters hemoglobin concentration and oxygen saturation and can, therefore, be measured in HSI [48]. Furthermore, changes in water content (peaking at about 980 nm) and fat content (peaking at about 740 nm) also contribute to the spectral profile [25]. Specifically for esophagus and colon cancer, healthy mucosa showed less oxygenation than cancer and the water content of cancer was higher than the water content of healthy mucosa [31].

Tumor tissue, which tends to have a higher tissue blood flow than normal tissue, can be altered significantly after neoadjuvant therapy [49], which may, consequently, induce additional spectral changes. Light penetration in tissue depends on the wavelength and amount of light absorption. Most tissue, including mucosa, is a relatively weak absorber, leasing to strongly diffuse reflectance and a significant depth penetration in the visible and near infra-red spectra. Penetration is, therefore, governed mainly by the wavelength and tissue type. Typical optical penetration in tissue is up to a few mm, with higher penetration in the NIR range [50]. Thus, the ability to use HSI to differentiate healthy and cancerous mucosa by HSI is restricted to tissue at this depth range. Further background on the spectral properties of tissue and cancer can be found in dedicated articles on this topic [25,51,52].

The presented ML models were trained using all spectral data from the camera (100 values corresponding to wavelengths in the range of 500 to 1000 nm at each pixel). Therefore, the ML models had full access to all available data to make a fair comparison. We recall that the 3DCNN model was trained with deep learning, and therefore, during the training process, it automatically learned the relevant spatio-spectral features contained in the hypercube required for classification in its network weights.

The performance of the 3DCNN may be harmed if we pre-process the spectra by, e.g., selecting certain spectral bands or using perfusion parameters, such as tissue water index (TWI) or St02 (which are derived from the original spectral data), as we may lose important information. In contrast, the ML models that do not involve deep learning (RBF-SVM and MLP) may benefit from specific pre-processing to supply them with relevant features. In future work, we aim to study this aspect to see if their performance can rival the 3DCNN. Furthermore, we aim to analyze the features learned by the 3DCNN using ‘explainable AI’ techniques [53], especially to determine the spectral bands that had the strongest influence on the prediction. This could be achieved using GradCAM [54].

We aimed to create a machine learning model that could recognize cancer automatically to avoid the need for a highly skilled human expert to be present in the OR. The same model could be used to help in the case of known and localized cancer to establish clear margins. However in this setting, we believe the model should be adapted or fine-tuned to incorporate data that is specific to the patient, using HSI data from the known cancer sites. This additional data will likely improve classification performance. It may be possible to combine approaches. Specifically, a general cancer recognition model, such as this work, could be used to detect cancer during the procedure, and it could then be adapted to the patient’s specific cancer in order to achieve precise margin prediction. We aim to develop such an approach in future work.

The results of our study showed that the combination of datasets from different anatomic regions could well improve the performance of the classification. The combination of the both datasets increased the ROC-scores, especially for esophagusgastric cancer, using the MLP (0.09 higher AUC) and RBF-SVM (0.11 higher ROC-AUC) models. Only the RBF-SVM improvement was found to be statistically significant (*p* = 0.0046). A larger dataset is required to have greater statistical power and validate if the performance improvements found with the esophagogastric patients are statistically significant with other models.

We believe that the findings of this study will motivate follow-up research to further explore combining data from different types of healthy and cancer tissue, to combat the pervasive challenges associated with acquiring large datasets. Especially in the medical field, it is still a problem to annotate tissue, since a precise annotation would require an extensive histopathological mapping to ensure the accuracy of the ground truth. However, this is incompatible with the clinical practice. Hence, combining datasets could help to achieve a robust classifier. In hyperspectral imaging, a high inter-patient variability of the spectra is a drawback to classify tissue correctly.

Consequently, largeer amounts of data from different patients are necessary to train a robust classifier. Using several anatomical regions with similar tissue types could facilitate data acquisition and help to acquire larger scale training datasets. The performance benefit that we observed might be explained in the context of multi-task learning (MTL). In MLT, a model is trained to perform different, yet related tasks simultaneously using training datasets specific to each task. The additional training data from the different tasks can help to prevent overfitting when data is limited, and to also learn useful model parameters that can be reused for the tasks [55].

In our case, we trained models to solve a combined task of differentiating healthy intestinal mucosa from adenocarcinoma tissue (cancer arising from the epithelial tissue of the intestinal mucosa). The large improvement in the RBF-SMV and MLP models by combining datasets suggests that they learned to generalize better using training samples from different mucosal regions (colon, esophagus and stomach). The benefit of combining healthy and cancer tissue datasets for training more robust classifiers could have a significant impact in the future to reduce the burden of collecting large datasets with HSI cameras specific to to particular cancer types. We aim to investigate if this effect is present in further studies to combine less similar tissue types, such as the liver and brain.

In our study, the trained model of the esophagogastric set was tested to classify colon cancer and achieved an ROC-AUC score of 0.67. The trained model of the colon dataset was tested to classify esophagogastric cancer. A higher ROC-AUC score was achieved with 0.74. As a result, the colon model seems to be more robust for the classification of cancer and healthy mucosa. We assume that the lower performance of the models are caused by the missed spectra of the tested tissue. Consequently, the combination of datasets from several tissue structures is an important step to achieve a high quality performance of models, which can be used in clinical practice. In future studies, more patients have to be included. Additionally, an enhancement of the models can be achieved by using multitask learning (MTL) approaches. MTL can improve outcomes by sharing knowledge among related tasks [56]. This approach can be proven in further studies.

Gastric cancer was previously detected in a dataset of 14 patients [57] with a reported accuracy of 79%. Multispectral imaging has been combined with various machine learning models to recognize gastric cancer [10]. The compared models include SVMs with linear and Gaussian kernels, Ada-Boost, RobustBoost and Random Forest-walk. The best algorithms were RobustBoost and linear SVMs, where RobustBoost achieved an averaged AUC of 70. In contrast to [57], LOPOCV evaluation has been performed [10]. Furthermore, it was mentioned that the inter-patient variability had a high impact on the results, and the ROC-curves of the RB showed a high variance.

In our study, a LOPOCV was also implemented, and we achieved a higher ROC-AUC of at least 0.09 higher than [10]. Since the datasets are different, it is impossible to categorically pinpoint the reason for the performance improvement, which could be a combination of the broader wavelength range used by us (from 500 to 1000 nm), higher quality images and more advanced machine learning models, in particular 3DCNNs as compared to SVMs used in Hohmann et al. [10]. The authors in Hohmann et al. [10] assumed that the mucus and the inflammation had an influence on the classification results.

Colon cancer detection was described using a quadratic SVM by [9], and an SVM with unknown kernel were used by [30]. Ref. [9] achieved an AUC of 0.87 and a MCC of 0.59 in a wavelength range from 400 to 1000 nm with 32 patients using simple cross validation. In our study, higher performance scores were achieved with LOPOCV. The clearly higher AUC scores especially for esophagus cancer detection by using the 3DCNN showed that this kind of method succeeds better in the case of large inter and intra-patient variability. Recent studies [58,59] showed that the inclusion of spatial information improved the accuracy especially in the case of 3DCNNs [60].

The 3DCNN model showed the highest ROC-AUC score in all evaluation studies, whereby the difference between the 3DCNN and MLP or RBF-SVM was less important for the colon dataset (the difference was 0.04 ROC-AUC). Additionally, the 3DCNN and RBF-SVM showed a more constant ROC-AUC score over all patients as opposed to the MLP model. We assume that that the spatial information used by the 3DCNN can analyze the spectral information more accurately and robustly than the other models, in addition to its ability to automatically learn relevant hierarchical spatio-spectral features.

The 3DCNN classifies each pixel in the hypercube using a 5 × 5 spatial window surrounding the pixel. Therefore, unlike the MLP and RBF-SVM models, the 3DCNN incorporates spatial context information. As the window is relatively small, the spatial context information is restricted to neighbouring pixels. Its main purpose is to provide better robustness to signal noise (because spectra at neighbouring pixels tend to be highly correlated) and to reduce errors made at pixels with strong specular reflections. In future research, we aim to investigate the value of using greater spatial context information. On the one hand, a larger spatial window would allow more spatial context information, e.g., to include spatial patterns associated with the tumor borders as features that can be automatically learned by the 3DCNN.

However, a larger spatial window would increase the number of trainable weights in the 3DCNN and may consequently lead to overfitting and worse generalization performance. Furthermore, in a real clinical setting, the surgeon may only see a small portion of the tumor, which limits the value of larger spatial context information. An interesting follow-up study would involve identifying the optimal amount of spatial context information by varying the window size (a hyper-parameter of the 3DCNN model). This could be implemented by optimizing the window size using a validation set (a part of the training dataset that is held out from training and used to automatically establish the optimal window size).

There are several factors that may contribute to inter-patient variability. Cancer stage variability and pre-therapy especially chemotherapy may be important factors. In our study, we used patients with several tumor T-grades and pre-therapies. The T-grade defines the extent of the tumor (e.g., the size and penetration depth). For example, T4 defined the highest infiltration into the tissue and a large size. Due to the light interaction with the tissue, different spectra for T-grades of tumors may be present. For example, with a superficial tumor with low penetration depth (T1), the light may pass through the tumor tissue and healthy mucosa. In contrast, with a high penetration depth (T4) the light may pass only through tumor tissue.

We aim to study spectral differences according to tumor grade in follow-up work, which would require a substantially larger dataset. Furthermore, pre-therapy can influence the tissue and its chemical components. Inter-patient variability can also be caused by the biological variability of human tissue and measurement noise of the HSI system. The Signal-to-Noise Ratio (SNR) of the HSI system was calculated in [26] with values between 30 and 50 dB.

## 4. Conclusions

In this work the 3DCNN model achieved a more accurate performance than classical machine learning models (MLP and RBF-SVM) to detect esophagogastric and colon cancer. Despite the small sample size, the results of this study are promising. We demonstrated that the MLP and RBF-SVM models can achieve substantially better results by combining datasets. However, the performance of the 3DCNN model was not improved by combining datasets, which goes against the general assumption that CNNs require more data to train as compared to the other methods. This can partially be explained by the fact that we used a relatively shallow 3DCNN with substantially fewer parameters (32,232) compared to CNNs used for image classification for instance, which may be of the order of millions. In future studies, we will aim to test whether deeper 3DCNNs also benefit from the additional training data by combining datasets.

The clinical application of this support technology is still not yet ready due to the relatively low DICE and MCC scores; where, when using a fixed decision threshold, they are below 60%. This is likely to be inacceptable for clinical use. There are two paths forward. The first path is to study the improvement in classification performance with more data and study whether the benefit of combining datasets from different cancer/tissue types is apparent with larger datasets. The second path stems from our observation that the DICE and MCC scores of all classification models were substantially improved using a patient-specific decision threshold.

This aspect has not been discussed in previous related works in HSI-based cancer classification with machine learning where fixed decision thresholds have been used. This finding provides an opportunity to improve the results with decision threshold fine-tuning at the test time. Its implementation would require the integration of additional medical knowledge. For instance, this could be achieved with an interactive software tool where a surgeon can tune the decision threshold so that classification results match the surgeon’s existing knowledge about the tissue characteristics that they have already acquired during the procedure.

This would use visual feedback similar to images shown in Figure 3 as a function of the decision threshold. For future research, we will conduct a study to evaluate whether surgeons can tune the detection thresholds with this approach in order to improve the classification performance.

## Figures and Tables

**Table 1:** Summary statistics of the colon dataset. There is one hypercube per patient. Each pixel corresponds to 100 hypercube voxels. These voxels give the relative reflectance at 100 wavelengths for a given pixel. Patients 2 and 6 did not have cancer.

Patient	Age	Gender	Proportion of Healthy Tissue Pixels (%)	Proportion of Cancer Tissue Pixels (%)	Tissue Location	T Classification	Tumor Grade
**1**	67	f	93.57	6.43	colon	pT3b	G2
**2**	68	m	100	0	colon	adenoma	
**3**	52	m	91.27	8.73	colon	pT2	G2
**4**	67	f	98.68	1.32	colorectal	pT2	G2
**5**	80	m	92.55	7.45	colorectal	pT2	G2
**6**	80	m	100	0	colon	adenoma	
**7**	81	m	56.16	43.84	colorectal	ypT3	G1 (mainly cancer)
**8**	66	m	97.12	2.88	colorectal	rpT2	G2
**9**	66	m	94.62	5.38	colorectal	ypT2	G1 (mainly cancer)
**10**	60	m	90.50	9.50	sigma	ypT3	G3
**11**	79	f	89.63	10.37	ascending colon	pT3	G2
**12**	59	m	96.59	3.41	colon ascendens	pT2	G2
**Mean**			91.72	8.28			

**Table 2:** Summary of the esophagogastric dataset. There is one hypercube per patient. AC, SC and G-E stand for adenoma carcinoma, squamous cell carcinoma and gestro-esophageal, respectively.

Patient	Age	Gender	Proportion of Healthy Stomach Tissue Pixels (%)	Proportion of Healthy Esophagus Tissue Pixels (%)	Proportion of Cancer Tissue Pixels (%)	Tumor~ Type	Tissue Location	T Classification
**13**	83	m	39.65	36.16	24.19	Not determined	G-E junction	ypT0
**14**	71	m	0	96.38	3.62	AC	G-E junction	ypT3
**15**	72	m	31.77	61.88	6.35	AC	G-E junction	ypT1b
**16**	67	m	60.59	31.11	8.30	AC	G-E junction	ypT1b
**17**	73	m	32.71	48.31	18.98	AC	G-E junction	ypT0
**18**	67	m	6.79	87.02	6.20	AC	G-E junction	ypT3
**19**	54	m	36.66	62.15	1.19	AC	G-E junction	ypT1b
**20**	65	f	99.13	0	0.87	AC	G-E junction	pT1b
**21**	56	m	61.64	16.31	22.06	AC	G-E junction	ypT2
**22**	60	m	30.34	67.09	2.57	AC	G-E junction	ypT0
**Mean**			39.93	50.64	9.43			

**Table 3:** ROC-AUC performance of different models evaluated on the colon dataset.

	Train Dataset: Colon, Test Dataset: Colon			Train Dataset: Combined, Test Dataset: Colon		
**Patient ID**	**RBF-SVM**	**MLP**	**3DCNN**	**RBF-SVM**	**MLP**	**3DCNN**
1	0.97	0.98	1.0	0.98	0.98	0.99
2	/	/	/	/	/	/
3	0.93	0.96	0.96	0.93	0.97	0.85
4	0.98	1.0	0.99	0.98	1.0	0.87
5	0.86	0.87	0.95	0.87	0.93	0.94
6	/	/	/	/	/	/
7	0.69	0.66	0.89	0.78	0.87	0.72
8	0.56	0.77	0.93	0.67	0.75	0.96
9	0.95	0.90	0.99	0.94	0.74	0.99
10	0.91	0.92	0.77	0.78	0.68	0.89
11	0.98	0.98	0.93	0.98	0.99	0.98
12	0.94	0.88	0.88	0.89	0.90	1.0
Mean ± S.D.	0.88 ± 0.12	0.89 ± 0.11	0.93 ± 0.069	0.88 ± 0.11	0.88 ± 0.12	0.92 ± 0.088

**Table 4:** ROC-AUC performance of different models evaluated on the esophagogastric (EG) dataset.

	Train Dataset: EG, Test Dataset: EG			Train Dataset: Combined, Test Dataset: EG		
**Patient ID**	**RBF-SVM**	**MLP**	**3DCNN**	**RBF-SVM**	**MLP**	**3DCNN**
13	0.96	0.96	0.91	0.98	0.99	0.99
14	0.81	0.85	0.99	0.99	0.98	0.98
15	0.91	0.92	0.92	0.87	0.92	0.95
16	0.91	0.87	0.98	0.91	0.96	0.96
17	0.59	0.67	0.89	0.73	0.48	0.89
18	0.53	0.47	0.90	0.80	0.87	0.85
19	0.81	0.93	0.93	0.95	0.99	0.97
20	0.68	0.55	0.71	0.85	0.77	0.79
21	0.91	0.94	0.92	0.99	1.0	0.96
22	0.80	0.79	0.99	0.95	0.98	0.98
Mean ± S.D.	0.79 ± 0.15	0.80 ± 0.17	0.91 ± 0.081	0.90 ± 0.082	0.89 ± 0.124	0.93 ± 0.067

**Table 5:** ROC-AUC performance of the MLP and 3DCNN models with dataset cross-training.

Train Dataset: EG, Test Dataset: Colon			Train Dataset: Colon, Test Dataset: EG		
**Patient ID**	**MLP**	**3DCNN**	**Patient ID**	**MLP**	**3DCNN**
1	0.64	0.96	13	0.80	0.99
2	/	/	14	0.75	0.77
3	0.34	0.59	15	0.91	0.83
4	0.67	0.61	16	0.84	0.79
5	0.64	0.90	17	0.72	0.68
6	/	/	18	0.48	0.78
7	0.79	0.43	19	0.82	0.99
8	0.59	0.89	20	0.49	0.90
9	0.57	0.92	21	0.84	0.95
10	0.87	0.58	22	0.71	0.94
11	0.65	0.98	-	-	-
12	0.91	0.96	-	-	-
Mean ± S.D.	0.67 ± 0.16	0.78 ± 0.20		0.74 ± 0.15	0.86 ± 0.11

**Table 6:** Matthews correlation coefficient (MCC) performance of different models evaluated on the colon and esophagogastric (EG) datasets.

Mean MCC ± S.D.	Patient-Generic Decision Threshold			Patient-Specific Decition Threshold		
	**RBF-SVM**	**MLP**	**3DCNN**	**RBF-SVM**	**MLP**	**3DCNN**
Train dataset: colon, test dataset: colon	0.37 ± 0.22	0.22 ± 0.26	0.49 ± 0.22	0.57 ± 0.31	0.53 ± 0.25	0.58 ± 0.23
Train dataset: combined, test dataset: colon	0.35 ± 0.23	0.29 ± 0.24	0.42 ± 0.16	0.57 ± 0.31	0.53± 0.28	0.55 ± 0.20
Train dataset: EG, test dataset: EG	0.27 ± 0.27	0.26 ± 0.26	0.41 ± 0.18	0.39 ± 0.30	0.34 ± 0.26	0.60 ± 0.25
Train dataset: combined, test dataset: EG	0.37 ± 0.23	0.33 ± 0.22	0.41 ± 0.22	0.63 ± 0.28	0.54 ± 0.29	0.51 ± 0.25

**Table 7:** DICE performance of different models evaluated on the colon and esophagogastric (EG) datasets.

Mean DICE ± S.D.	Patient-Generic Decision Threshold			Patient-Specific Decision Threshold		
	**RBF-SVM**	**MLP**	**3DCNN**	**RBF-SVM**	**MLP**	**3DCNN**
Train dataset: colon, test dataset: colon	0.39 ± 0.24	0.36 ± 0.22	0.50 ± 0.24	0.52 ± 0.25	0.58 ± 0.24	0.61 ± 0.24
Train dataset: combined, test dataset: colon	0.38 ± 0.24	0.32 ± 0.25	0.44 ± 0.18	0.56 ± 0.25	0.57 ± 0.28	0.59 ± 0.20
Train dataset: EG, test dataset: EG	0.30 ± 0.29	0.29 ± 0.26	0.41 ± 0.20	0.49 ± 0.31	0.38 ± 0.26	0.62 ± 0.26
Train dataset: combined, test dataset: EG	0.38 ± 0.25	0.34 ± 0.24	0.40 ± 0.13	0.56 ± 0.30	0.60 ± 0.24	0.52 ± 0.26

## Data Availability

The data underlying the results presented in this paper are not publicly available at this time but may be obtained from the authors upon reasonable request.

## Abbreviations

The following abbreviations are used in this manuscript:

ML	Machine Learning
SVM	Support Vector Machine
RBF-SVM	Radial basis function Support Vector Machine
MLP	Multi-Layer Perceptron
CNN	Convolutional Neural Network
3DCNN	Three Dimensional Convolutional Neural Network
SNV	Standard Normal Variate
HSI	Hyperspectral imaging
LOPOCV	Leave-one-patient-out cross-validation
EG	esophagogastric
